# Comparison of mammographic findings and need for ultrasound and biopsy in women undergoing wide local excision and either intraoperative radiotherapy or external beam whole breast irradiation

**DOI:** 10.1186/bcr2966

**Published:** 2011-11-04

**Authors:** A Romsauerova, J Dewar, D Brown, A Evans

**Affiliations:** 1 Dundee Cancer Centre, University of Dundee, UK

## Introduction

TARGIT-A is a recent prospective randomised multicentre controlled trial comparing intraoperative radiotherapy (IORT) and external beam whole breast irradiation (EB). The aim of this study was to compare localised and generalised findings at follow-up mammography and the need for interventions such as ultrasound and biopsy between women in the two treatment arms from a single recruiting centre.

## Methods

We have compared the 61 women who received IORT alone with the 63 women who received EB alone. All mammograms were reviewed by radiologists blinded to the treatment received. The focal soft tissue appearance was classified. The presence of generalised skin thickening and increased density was recorded. The performance of ultrasound and/or biopsy was recorded.

## Results

The number of follow-up mammograms and length of follow-up was similar in both groups (2.46 IORT vs. 2.09 EB and 3.27 years IORT vs. 3.0 years EB). There was no difference in the mammographic appearance of the postoperative site between the two groups. However, generalised skin thickening and increase in density were more common in the EB group compared with the IORT group (20 of 63 (37%) vs. 10 of 61 (16%), *P *= 0.04 and 20 of 63 (37%) vs. 5 of 61 (8%), *P *= 0.001, respectively). Ultrasound at follow-up was more frequent in the IORT group compared with the EB group (15 of 61 (25%) vs. 7 of 63 (11%), *P *= 0.049).

## Conclusion

Generalised reactions on mammography are more common following EB compared IORT. However, follow-up ultrasounds were more frequent in the IORT group.

